# Prehabilitation in Obese Patients with Ventral Hernia: A Narrative Review and Proposal of a Clinical Algorithm

**DOI:** 10.3390/jcm15082942

**Published:** 2026-04-13

**Authors:** Monika Maćków, Grzegorz Sęk, Michaela Godyla-Jabłoński, Ewa Raczkowska, Marek Zawadzki, Katarzyna Neubauer

**Affiliations:** 1 Department of Human Nutrition, Faculty of Biotechnology and Food Science, Wrocław University of Environmental and Life Sciences, 37 Chełmońskiego Street, 51-630 Wrocław, Poland; monika.mackow@upwr.edu.pl (M.M.); michaela.godyla@upwr.edu.pl (M.G.-J.); 2 Outpatient Clinic of Oncological Surgery, Regional Specialist Hospital in Wrocław, Research and Development Centre, Kamieńskiego 73A, 51-124 Wrocław, Poland; 3 Department of General and Minimally Invasive Surgery with Subdivisions of Metabolic and Endocrine Surgery, Regional Specialist Hospital in Wrocław, Research and Development Centre, Kamieńskiego 73A, 51-124 Wrocław, Poland; grzegorz.sek1@gmail.com (G.S.); or marek.zawadzki@pwr.edu.pl (M.Z.); 4 Department of Oncological Surgery, Regional Specialist Hospital in Wrocław, Research and Development Centre, Kamieńskiego 73A, 51-124 Wrocław, Poland; 5 Department of Procedural Clinical Sciences, Faculty of Medicine, Wrocław University of Science and Technology, Hoene-Wrońskiego 13 c, 58-376 Wrocław, Poland; 6 Department of Gastroenterology, Hepatology and Internal Medicine, Wrocław Medical University, Borowska 213, 50-556 Wrocław, Poland; katarzyna.neubauer@umw.edu.pl

**Keywords:** preoperative optimization, hernia, obesity, surgical site infection sarcopenia, smoking, alcohol, physical activity, dietary intervention, omega-3 fatty acids

## Abstract

Background: Overweight and obesity are major health problems of the 21st century. As a significant risk factor for numerous noncommunicable diseases, obesity is also strongly associated with the development of abdominal hernias, which significantly impair patients’ quality of life. The review focuses on the pathophysiological mechanisms linking obesity to hernias and the impact of key prehabilitation components. Available research indicates a complex interrelationship between obesity and the development of ventral hernias, driven by pathophysiological mechanisms such as increased intra-abdominal pressure and chronic inflammation, which weakens the collagen matrix of the abdominal wall. Furthermore, both smoking and alcohol consumption significantly increase the risk of abdominal obesity and surgical complications; in turn, physical activity is crucial for reducing visceral fat. Psychological support may reduce pre-operative stress and contribute to improved outcomes. Nutritional intervention and weight loss are other essential components of preoperative management for ventral hernia repair. This review aims to highlight the role of prehabilitation in ventral hernia surgery in obese patients and to propose a structured, evidence-based algorithm (DEPP) for this high-risk population. The algorithm includes: Dietary intervention (D), Elimination of smoking and alcohol consumption (E), Physical activity (P), and Psychological support (P). The algorithm was developed to systematize the clinical approach and determine the steps to be taken in the treatment of patients with obesity and abdominal hernia. Methodology: A literature search was conducted across PubMed, Scopus, and Google Scholar databases for articles published between 2002 and 2026. We included randomized controlled trials, prospective/retrospective cohort studies, systematic reviews, and meta-analyses. Conclusions: Prehabilitation is a multifaceted strategy for optimizing the health of patients with obesity prior to abdominal hernia repair. The proposed prehabilitation algorithm, known as DEPP, is a preliminary approach for managing this group of patients.

## 1. Introduction

**Figure 1:**
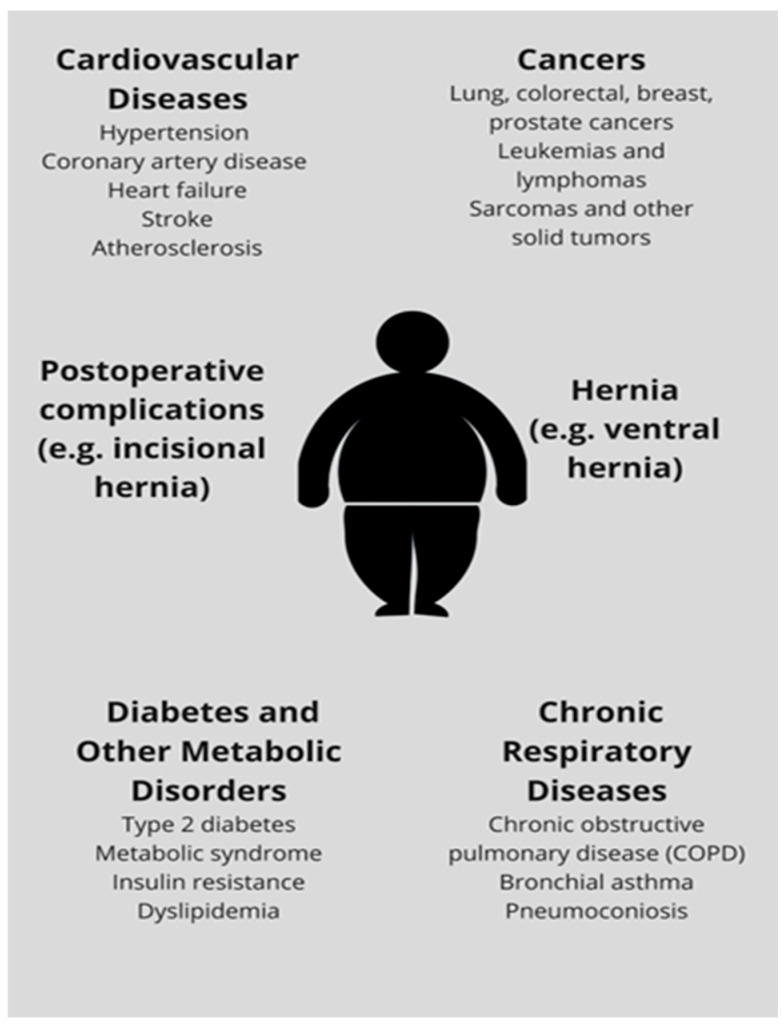
Major non-communicable diseases associated with obesity.

According to the European Hernia Society (EHS), ventral hernias are defined as defects in the abdominal wall fascia that are neither inguinal nor hiatal. This definition encompasses both primary and incisional hernias located in the anterior abdominal wall [1]. Ventral hernias (VHs) are common abdominal wall defects that pose a significant surgical challenge due to their high recurrence rate and associated postoperative complications [2]. Incisional hernias are a potential complication following abdominal surgery. It is estimated that up to 30% of patients undergoing open abdominal surgery will develop an incisional hernia (IH) within two years of surgery [3]. In this review, the term “ventral hernia” is used as an umbrella term encompassing both primary and incisional hernias, unless otherwise specified. Abdominal hernias are an increasingly common surgical condition, and the most important risk factors include: overweight or obesity, age over 40 years, male gender, previous surgery, abdominal trauma, multiparity [4,5,6], chronic cough, and smoking [6]. Obesity is strongly associated with multiple non-communicable diseases, as illustrated in Figure 1.

A new obesity classification was published in 2025, distinguishing between preclinical and clinical obesity. Preclinical obesity is defined as an excess amount of adipose tissue with preserved organ and tissue function. Although asymptomatic, preclinical obesity is associated with an increased risk of progression to clinical obesity. In contrast, clinical obesity is characterised as a chronic systemic disease involving dysfunction of tissues, organs, or entire physiological systems resulting from excessive adiposity. It may lead to organ damage, impaired physiological functions, and life-threatening events such as myocardial infarction, stroke, and renal failure [7].

Excess adipose tissue is associated not only with an increased risk of developing various types of hernias, but also an increased risk of surgical complications, including increased hernia recurrence and postoperative complications [8]. Prehabilitation in patients with obesity and concomitant ventral hernia is a multifaceted approach. It encompasses weight reduction strategies, including pharmacotherapy with GLP-1 receptor agonists or bariatric procedures, as well as nutritional optimisation, psychological support, and individually tailored physical activity programmes. Such an approach is justified by the critical importance of optimising the patient’s condition prior to elective repair. In this population, prehabilitation plays a key role in reducing the risk of postoperative complications [2].

Despite well-documented risk factors associated with ventral hernia repair, there is currently a lack of standardized prospective studies and uniform preoperative protocols specifically designed for this patient population. This limits the development of comprehensive guidelines on key elements of post-operative prehabilitation. At the same time, post-operative rehabilitation is increasingly recognized as an essential strategy for reducing perioperative risk and improving post-operative outcomes. Therefore, the aim of this study was to propose a structured post-rehabilitation algorithm to optimize clinical management and improve long-term outcomes in patients undergoing ventral hernia repair.

## 2. Methodology

### 2.1. Study Design and Search Strategy

This study is a comprehensive narrative review utilizing a systematic search strategy. A literature search was conducted across PubMed, Scopus, and Google Scholar databases for articles published between 2002 and 2026. The keywords used in the search included abdominal hernia; ventral hernia; obesity; prehabilitation; smoking; alcohol; physical activity; dietary intervention; psychological support; omega-3 fatty acids; vitamin D; inflammation; abdominal wall repair; surgical site infection; visceral adipose tissue; surgical treatment.

### 2.2. Inclusion and Exclusion Criteria

Studies were included if they met the following criteria: (1) published in English or Polish; (2) involved adult patients (≥18 years) undergoing elective ventral or incisional hernia repair; (3) evaluated preoperative optimization (DEPP algorithm components). We included randomized controlled trials, prospective/retrospective cohort studies, systematic reviews, and meta-analyses. We excluded case reports, editorials, and studies focusing solely on surgical techniques without preoperative optimization data.

### 2.3. Data Selection and Extraction

The initial search yielded 527 results. A total of 270 articles were excluded because they focused on intraoperative techniques, pediatric patients, other types of hernias, or did not include clinical outcome data in the context of post-rehabilitation. Discrepancies in article selection were resolved by consensus.

A final set of 257 articles was selected for this review.

The landmark studies identified during the literature review, which form the clinical basis for each step of the DEEP algorithm, are summarized in Table 1. The table includes study designs, sample sizes, primary clinical outcomes, and the corresponding Levels of Evidence (LoE).

## 3. Pathophysiological Mechanisms Linking Obesity to Ventral Hernia

### 3.1. Obesity and Visceral Adiposity as Risk Factors for Ventral Hernia Development

The development of abdominal hernias and the accumulation of adipose tissue are consequences of obesity within the abdominal wall [8,15]. There are two main types of adipose tissue with distinct metabolic functions: subcutaneous adipose tissue (SAT) and visceral adipose tissue (VAT). Excessive accumulation of VAT surrounding the intra-abdominal organs is commonly referred to as visceral or central obesity. This condition is strongly associated with a number of metabolic disorders, including impaired glucose and lipid homeostasis, insulin resistance, and an increased risk of cardiovascular disease, hypertension, coronary artery disease, and certain malignancies [16,17,18]. One of the key risk factors for ventral hernia (VH) is the volume and distribution of adipose tissue, particularly visceral fat. Higher visceral fat content in the abdominal cavity chronically increases intra-abdominal pressure (IAP) by exerting pressure on the abdominal and pelvic cavities, which is the primary mechanism contributing to hernia formation [19,20].

Large population-based studies provide additional evidence for this association. In one of the largest studies, data on single-nucleotide polymorphisms (SNPs) associated with abdominal hernia (AH) were obtained from the FinnGen Biobank (FB), while SNPs associated with BMI were obtained from the UK Biobank (UKB). The study demonstrated a genetic association between BMI and AH, showing that the risk of developing AH increases proportionally with BMI [21]. Similarly, a genome-wide association study in a European population showed that increased BMI, body fat percentage, fat mass, visceral fat, waist circumference, and hip circumference were causally associated with a higher risk of ventral hernia [22]. Lau et al. [23], in a multicentre study involving a total of 2,807,414 patients from 14 hospitals, provided strong clinical evidence that a BMI above 40 kg/m^2^ may indicate an increased risk of incarceration, while a BMI exceeding 60 kg/m^2^ represents the highest risk of incarceration of ventral hernias other than inguinal ones (*p* < 0.0001). Further studies in various populations have consistently confirmed the comorbidity of hernias and obesity [24,25,26,27,28], including ventral hernia [4,22,29,30,31]. It is also worth noting that common obesity-related comorbidities, such as lipid metabolism disorders and cardiovascular disease, metabolic syndrome, further contribute to the development of hernias [25,32,33].

Additional risk factors commonly associated with obesity include obstructive uropathy, chronic constipation, and chronic obstructive pulmonary disease [1,4,34]. Taken together, these results provide strong evidence that maintaining a healthy BMI can significantly reduce the risk of ventral hernias. The authors of these studies emphasise that physicians should remain vigilant regarding the increased risk of hernias in patients with obesity or a high BMI [28].

It is therefore important to emphasize that both the amount and distribution of body fat, especially visceral fat, play a significant role in the risk of developing a ventral hernia. Furthermore, complications of excess body weight may also constitute risk factors for ventral hernia. These relationships underscore the importance of comprehensive preventive care, aimed not only at reducing BMI but also at controlling the comorbidities associated with obesity.

### 3.2. Obesity as an Inflammatory Condition and the Risk of Abdominal Hernias

Obesity is recognised as a chronic low-grade inflammatory condition characterised by increased infiltration of adipose tissue by immune cells and elevated production of proinflammatory cytokines, including TNF-α and IL-6. This excess of proinflammatory cytokines and the chronic inflammation associated with obesity are involved in the development of obesity complications [35,36]. It is worth noting that elevated levels of C-reactive protein (CRP) are frequently observed in obese individuals and are associated with indicators such as BMI and waist-to-hip ratio (WHR). An analysis of 127 patients with complex abdominal hernias, conducted by Geletzke et al. [35], confirmed significant associations between BMI, CRP levels, and antioxidant deficiency, in this case zinc. Zinc deficiency can result in chronic inflammation, disrupt the balance of trace elements, and significantly impact postoperative wound healing. Similar results were obtained in studies [36,37,38,39]. Currently, a growing body of evidence proves that inflammation itself plays a significant role in the development, progression, and postoperative healing of ventral hernias. Cytokines and related inflammatory pathways may be key elements in its pathogenesis, in part through their influence on metabolic disturbances in connective tissue. For instance, a study by Yaqin Qi et al. [40] identified cytokines involved in the pathogenesis of ventral hernias using Mendelian randomisation. In this study, obesity was associated with the overactivation of specific proinflammatory cytokines, such as TNF-α, which inhibit the synthesis and expression of type I collagen a key structural component of connective tissue. This process disrupts the organisation of collagen fibres, reduces their thickness, and weakens the mechanical properties of the extracellular matrix of the abdominal wall. Other studies have also confirmed that the development of abdominal hernias in obese individuals is associated with impaired collagen metabolism, manifested by a reduced ratio of collagen type I to type III [41,42,43].

These results indicate that chronic inflammation and impaired collagen metabolism in obesity contribute not only to hernia formation but also to impaired wound healing and an increased risk of recurrence.

These mechanisms underscore the importance of targeting inflammatory pathways and connective tissue integrity as part of comprehensive preoperative optimization.

Further high-quality research is needed to further define the clinical impact of these processes and develop appropriate strategies to improve surgical outcomes in obese patients undergoing ventral hernia repair.

### 3.3. Sarcobesity and the Risk of Ventral Hernia

Sarcopenia is defined as a decline in muscle mass and strength that progresses with age [44,45]. In addition to age-related changes, catabolic processes and muscle atrophy can also be induced by other physiological or pathological conditions, such as immunosuppression, liver cirrhosis, trauma, prolonged immobilisation, or malignant tumours [44]. Importantly, sarcopenia can coexist with obesity, giving rise to a newly recognised clinical entity called sarcopenic obesity (SO) [45,46,47].

Sarcopenic obesity is a condition in which the amount of body fat increases and muscle mass gradually decreases with age. It is accompanied by chronic inflammation and metabolic disorders, which often coexist with obesity. This syndrome, in turn, contributes to long-term adverse health effects [48]. In SO, muscle function is impaired due to the presence of increased amounts of adipose tissue, which may lead to weakening of the abdominal walls, thus increasing the risk of developing, for example, an incisional hernia [49,50,51].

There is currently a debate in the literature regarding the association between ventral hernia and sarcopenia. In a study of 148 patients undergoing complex ventral hernia repair, Rinaldi et al. [47] observed sarcopenia in 26% of patients, including 20% with obesity-related sarcopenia. However, the results of this study showed that obesity may mask sarcopenia findings and that sarcopenia and abdominal muscle mass indices are not reliable predictors of biological abnormalities or recovery after hernia repair. Similar results were demonstrated in a study by Siegal et al. [44] in a group of 135 patients and by van Rooijen et al. [49] in a group of 283 patients, in which no significant association between sarcopenia and incisional hernia development was found. These results show that sarcopenia is not a clearly significant risk factor for abdominal hernia development. However, other studies have shown a significant association between hernia development and obesity. In a study by Takano et al. [52], conducted on 262 patients who underwent laparoscopic resection of colorectal cancer, the development of incisional hernia was significantly associated with subcutaneous obesity (*p* = 0.002), visceral obesity (*p* = 0.002), sarcopenia (*p* < 0.001), and wound infection (*p* < 0.001). Sarcopenia and wound infection were also found to be independent risk factors for hernia. The highest odds ratio was observed in cases with the coexistence of visceral obesity and sarcopenia. Similarly, a study by Bailey et al. [53] found that sarcopenia more than tripled (odds ratio ≈ 3.3) the risk of developing an incisional hernia after abdominal cancer surgery. The authors of this study emphasised the importance of preoperative optimisation of physical activity and nutritional status.

Published data indicate a possible association between sarcopenia, sarcopenic obesity, and the risk of developing ventral and incisional hernias. However, currently available scientific evidence remains limited. Significant heterogeneity across studies, the lack of standardized and up-to-date definitions of sarcopenia, and small sample sizes in many analyses undermine the consistency and reliability of available results, limiting their interpretation and clinical application [54]. Therefore, well-designed prospective multicenter studies using standardized definitions of sarcopenia consistent with the ESPEN guidelines are necessary to precisely determine its role in the pathophysiology of ventral hernias.

**Figure 2:**
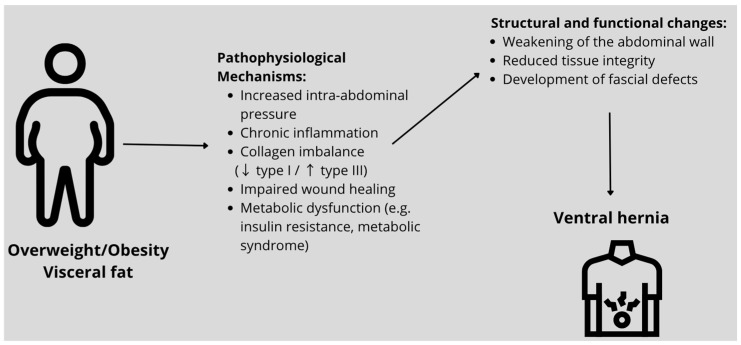
Risk factors and mechanisms influencing the development of ventral hernia in people with obesity. ↑: increased; ↓: decreased.

Risk factors and mechanisms influencing the development of ventral hernia in obese individuals are presented in Figure 2.

## 4. Surgical Treatment of Ventral Hernias and Postoperative Complications in Obese Patients

### 4.1. Surgical Management of Ventral Hernias in Obese Patients

In recent years, the dynamic development of surgical techniques for abdominal hernias has led to the emergence of abdominal wall surgery as a distinct and highly specialised field of medicine. Currently, the management of abdominal wall defects encompasses open approaches, minimally invasive techniques, and component separation techniques (CST) [3].

#### 4.1.1. Surgical Strategies for Ventral Hernia Repair

Modern surgical repair of hernias typically combines autologous tissue closure with reinforcement using synthetic mesh. This mesh gradually becomes incorporated into the fascia through fibrosis in the postoperative weeks [55].

The Rives–Stoppa technique remains the gold standard for open surgical repair of abdominal hernias. It provides durable outcomes and high efficacy, which largely stems from the placement of the mesh in the retromuscular space. This positioning allows broad coverage of the defect, prevents direct contact with intra-abdominal organs, and is consistently associated with lower recurrence rates, reduced incidence of wound infections, and improved functional stability of the abdominal wall [56]. A commonly performed example is the repair of a midline incisional hernia. In this procedure, the surgeon first opens the retrorectus space (the plane between the dorsal surface of the recti muscles and their posterior sheath), places the mesh in this position, and then closes the posterior and anterior sheath edges with sutures [3].

Minimally invasive techniques for ventral hernia repair include eTEP (enhanced-view totally extraperitoneal plasty), TARUP (transabdominal retromuscular umbilical prosthesis) and TAPP (transabdominal preperitoneal). Both TARUP and eTEP are retromuscular approaches that adapt Rives–Stoppa principles through laparoscopic or robotic platforms, thereby allowing for a significant reduction in postoperative pain [3,57], shorter hospital stays, and fewer complications [58]. However, these procedures require incision and subsequent reconstruction of the posterior rectus sheaths along the midline, which may affect the biomechanics of the abdominal wall [56].

During hernia repair, reapproximation of the defect edges necessitates repositioning of internal organs (mainly the intestines) into the abdominal cavity, which reduces the effective postoperative abdominal cavity volume [59]. This increases tension on the fascial edges. In the event of surgical failure, a new defect may arise, often larger than the original, while previously dissected anatomical planes become altered, complicating the possibility of performing a subsequent procedure in the same manner [60]. Therefore, achieving success at the first attempt is crucial.

All available techniques and adjuncts should be utilised according to the size and complexity of the hernia defect. For defects with a significant transverse dimension, BTA (botulinum toxin A) injections are often administered 4–6 weeks prior to surgery to relax and lengthen the lateral muscles, facilitating closure of the abdominal wall [61].

For large fascial defects requiring component separation, anterior component separation (external oblique release, EOR) and posterior component separation (transversus abdominis release, TAR) are commonly utilised. These manoeuvres function as a critical supplement to reduce tension on the fascial plane. Additionally, the TAR technique enables the surgeon to utilise a large mesh with an overlap extending across the midline to both lumbar areas. The choice of technique is determined by the surgeon and depends on hernia characteristics, patient anatomy, patient-related factors, and the type of repair performed [3].

#### 4.1.2. Bariatric Procedures in the Context of Ventral Hernia Surgery

Another key goal for patients with obesity and ventral hernia is preoperative weight loss [62]. According to the 2022 recommendations of the American Society for Metabolic and Bariatric Surgery (ASMBS) and the International Federation for the Surgery of Obesity and Metabolic Disorders (IFSO), in patients with severe obesity and ventral hernia requiring elective repair, bariatric procedures may be considered as a precursor to achieving significant weight loss, which may reduce postoperative complications and improve the durability of the hernia repair. This is particularly true for patients with a BMI > 35 kg/m^2^, as it provides effective and lasting weight loss. Obese patients are at increased risk of surgical site infections (SSIs) and other wound complications [63].

Therefore, bariatric procedures may be considered in obese patients, along with selecting an appropriate surgical technique to reduce postoperative complications and improve the patient’s quality of life. These challenges highlight the critical importance of preoperative optimization in obese patients undergoing ventral hernia repair.

### 4.2. Postoperative Complications After Ventral Hernia Repair in Obese Patients

Obesity is a significant risk factor for postoperative complications following ventral hernia repair (VHR), with the risk increasing alongside BMI, particularly above thresholds of 32, 40, and 50 kg/m^2^ [64,65,66,67]. Rodrigues-Gonçalves et al. [2] demonstrated a significantly higher complication rate in obese patients compared to non-obese individuals, even among those who achieved preoperative weight loss. In this cohort, hernia recurrence occurred in 23% of patients after weight reduction, while postoperative complications were observed in 37% of cases; in contrast, obese patients without prior weight loss had a complication rate of 33%. Postoperative wound complications (PWC) were the most frequent issues in obese patients, and the highest recurrence rate was observed among those who remained obese without weight reduction (14%).

Several meta-analyses have shown that obesity increases the risk of complications following open VHR, whereas laparoscopic and robotic approaches are associated with lower complication rates [68,69]. Similar findings were reported by Froylich et al. [70] in a study of patients with a BMI > 30 kg/m^2^ undergoing either laparoscopic (LVHR) or open ventral hernia repair (OVHR); postoperative complications occurred in 17.1% of the LVHR group compared to 20.5% in the open group. In the study by Willoughby et al. [71], open-surgery patients with a BMI of 25–30 kg/m^2^ had a higher rate of surgical site infections than overweight patients undergoing laparoscopic surgery (*p* = 0.026). Conversely, the study by Alizai et al. [72] did not find a significantly higher complication rate in obese versus non-obese patients (35% vs. 22%; *p* = 0.083), although operative time was longer for obese patients, particularly with the open technique.

One meta-analysis [73] of randomised controlled trials comparing laparoscopy with the open approach in patients with primary ventral hernias found that the overall recurrence rate was twice as low with the laparoscopic approach (RR = 0.49; 95% CI 0.32–0.74; *p* < 0.001; I^2^ = 29%). Local infections, wound dehiscence, and seroma occurred significantly less frequently in patients treated laparoscopically. Similar outcomes were reported by Lee et al. [74] in obese patients.

These findings suggest that laparoscopy provides superior perioperative outcomes in obese patients by reducing the risk of wound complications, shortening hospital stay, and lowering the risk of hernia recurrence. Both the International Endohernia Society and Italian guidelines recommend laparoscopic surgery for patients with a BMI ≥ 30 kg/m^2^ due to the reduced risk of wound complications and comparable long-term results [75,76].

Despite advances in surgical techniques and preoperative optimization, obese patients are at increased risk of postoperative complications. Therefore, appropriate preoperative management should be implemented to reduce the incidence of complications.

## 5. The Role of ERAS and Prehabilitation in Abdominal Hernia Surgery in Patients with Obesity

### 5.1. Implementation of ERAS Protocols in Obese Patients Undergoing Abdominal Hernia Repair

The Enhanced Recovery After Surgery (ERAS) protocol is a structured, multidisciplinary approach to perioperative care, encompassing the preoperative, intraoperative, and postoperative phases. Its primary goals are to minimize surgical stress, accelerate recovery, and reduce complications, hospital stay, and overall treatment costs [77].

The ERAS protocol has been used in abdominal surgery, including abdominal wall reconstruction. A study on large incisional hernia repair [78] showed a shorter hospital stay in the ERAS group (median 4 vs. 5 days; *p* < 0.001) without increased complications. Similar benefits were observed in a prospective study of 109 patients undergoing robotic VHR [57], including reduced length of stay, effective pain control, and low readmission rates. The authors also highlighted the importance of preoperative optimisation, including weight loss in obese patients These results support the importance of the ERAS protocol in robotic ventral hernia surgery and highlight its potential to further improve patient outcomes and shorten recovery times. Members of the Institutional Quality Improvement (QI) Committee of the Abdominal Core Health Quality Collaborative (ACHQC) reviewed 295 individual protocol elements from six referral centers specializing in ventral hernia repair. These elements included preadmission optimization, preoperative care, intraoperative management, and postoperative care. The review identified common elements that could form the basis for developing an even more standardized ERAS protocol for hernia repair, and future research will likely contribute to the development of formal guidelines for this surgical area [79]. In summary, implementing ERAS protocols for abdominal hernia surgery in obese patients significantly shortens hospitalization, improves pain control and bowel function, and reduces the risk of complications. Preoperative optimization, including weight loss and nutritional support, is an important element in improving safety and surgical outcomes in this group of patients.

ERAS protocols for ventral hernia repair in obese patients significantly shorten hospital stays and improve recovery, including pain control and bowel function. Prehabilitation, particularly weight loss and nutritional support, remains an important factor in reducing the risk of complications in these patients. However, the current lack of large randomized controlled trials poses a significant challenge in formulating guidelines. Further research is needed to standardize perioperative care in this high-risk group.

### 5.2. Prehabilitation—A Preoperative Preparation Programme

Prehabilitation is a comprehensive approach aimed at improving patients’ health and functional capacity before planned surgical or oncological treatment. It achieves this by modifying risk factors for complications, increasing physiological reserves, and improving the body’s tolerance to stress [80,81,82]. Many prehabilitation programmes described in the literature last from four to eight weeks. The preparatory period for surgery includes medical optimisation, physical exercises, nutritional and psychological support. These interventions are carried out by a multidisciplinary team consisting of surgeons, anaesthetists, geriatricians, physiotherapists, dietitians, and psychologists [82].

The importance of prehabilitation is increasingly recognised in international guidelines. In its 2021 recommendations, ESPEN highlighted the role of preoperative nutritional support and physical activity in reducing postoperative morbidity [83]. The same organisation’s 2025 recommendations now explicitly state that prehabilitation should be offered based on risk stratification [84], meaning that such programmes should be individualised rather than applied uniformly to all patients. Instead, the risk of postoperative complications should be assessed for each individual patient. Therefore, the ultimate goal of prehabilitation is to reduce the incidence and/or severity of complications, improve patient outcomes, and accelerate recovery [82].

A growing body of evidence indicates that prehabilitation, particularly in the context of oncological and other surgical procedures, reduces postoperative complications [85,86]. A study by Liang et al. [87] analysed obese patients preparing for inguinal hernia surgery. The inclusion of prehabilitation increased the percentage of patients without recurrence and postoperative complications (69.5% vs. 47.5%, *p* = 0.015).

Prehabilitation represents a novel, comprehensive, and multidisciplinary approach that improves perioperative outcomes by improving patients’ functional capacity and reducing the risk of complications. Current data, primarily from oncology and general surgery populations, suggest potential benefits of appropriately designed prehabilitation for other patients.

### 5.3. Multimodal Prehabilitation

Multimodal prehabilitation is defined as a combination of two or more intervention components selected for their potential cumulative or synergistic effect on health outcomes. Typical components include:

- aerobic and endurance exercise—to support cardiovascular function and strengthen the musculoskeletal system
- nutritional interventions—to prevent malnutrition, support anabolism, and meet increased metabolic demands,
- psychological support—to reduce stress and unhealthy behaviours
- medical optimization—treatment of anemia and other comorbidities, drug analysis [81].

The effectiveness of multimodal prehabilitation has been confirmed in numerous studies [88,89,90]. Furthermore meta-analyses of both randomised clinical trials and observational studies have demonstrated that these interventions shorten hospital stays, reduce the incidence of complications, and improve the overall quality of life [91,92].

One of the key arguments for implementing multimodal prehabilitation in medical facilities is its economic impact. Literature review [93], which included 45 studies, found that prehabilitation for patients awaiting elective surgery is cost-effective compared to standard preoperative care, particularly for oncology patients, patients with high perioperative risk, and shorter or home-based programs. However, the authors emphasised that these results should be interpreted with caution, as many of the included studies were at high risk of bias and/or had low methodological quality. Available evidence suggests that prehabilitation provides significant health benefits to patients while reducing hospitalisation costs.

Multimodal prehabilitation, combining health stabilization, exercise, nutritional support, and psychological interventions, has been shown to shorten hospital stays, reduce complication rates, and improve patient outcomes. Evidence also supports its cost-effectiveness, highlighting its potential value in preoperative care.

### 5.4. Prehabilitation Before Surgery for Ventral Hernia

Prehabilitation before ventral hernia repair is an increasingly common trend in modern surgery. Its main goal is to minimise risk factors and improve the patient’s overall health before ventral hernia surgery. One study of prehabilitation before ventral hernia repair, demonstrating a positive effect of prehabilitation in obese patients undergoing ventral hernia repair (VHR), was conducted by Liang et al. [87]. The prehabilitation group, which included nutritional counseling and physical activity, demonstrated a higher percentage of patients achieving weight loss and other preoperative goals. A lower risk of wound complications was observed, although this difference was not statistically significant (6.8% vs. 17.6%, *p* = 0.167). Furthermore, the likelihood of hernia recurrence and other complications was significantly lower in the prehabilitation group (69.5% vs. 47.5%, *p* = 0.015). Another Spanish study [2] included 165 patients undergoing ventral hernia repair. In this single-centre study, obese patients with ventral hernias also received prehabilitation. Postoperative complications were more common in patients diagnosed with obesity. However, postoperative outcomes were influenced not only by obesity but also by hernia complexity and preoperative risk profile, suggesting that weight loss alone may not be sufficient. Hernia recurrence was more common in patients without preoperative weight loss, although this difference was not statistically significant. In a Canadian study [94], 70 patients with ventral hernia underwent a multimodal prehabilitation programme, of whom 57.1% completed it. Participants showed improved functional capacity, and prehabilitation facilitated optimisation for surgery. These findings suggest that multimodal approaches are more effective than weight loss alone. Other studies confirm that prehabilitation benefits obese patients and those undergoing abdominal hernia surgery [95,96]. In contrast, Bernardi et al. [97] found no significant differences in long-term outcomes, including complications or hernia recurrence, between the prehabilitation group and the no prehabilitation group. The authors of this study concluded that prehabilitation is not always justified in obese patients undergoing elective VHR. Available evidence suggests that multimodal prehabilitation may benefit both patients and surgeons, especially if it is not limited solely to weight loss. Prehabilitation may also benefit older patients undergoing complex hernia repair, although evidence in this group remains limited.

In 2022, the European Hernia Society (EHS) published a systematic review [98], which demonstrated that the number of studies on prehabilitation prior to ventral hernia surgery remains limited and the available results are inconclusive. Consequently, most recommendations are based on expert consensus rather than high-quality evidence.

Available evidence suggests that prehabilitation, especially in its multimodal form combining nutritional interventions, physical activity, and psychological support, can reduce the risk of postoperative complications, shorten hospital stays, and improve patients’ quality of life. This may be due to methodological limitations, such as small sample size or high study dropout rates. The implementation of prehabilitation programmes remains limited due to variability in clinical practice and lack of standardisation, which may affect their accessibility and effectiveness. These limitations may affect both the accessibility of interventions and the standardisation and effectiveness of prehabilitation programmes, as demonstrated by Marker et al. [99]. The 2025 EHS recommendations [62] include lifestyle modification, the use of GLP-1 analogues, and, in patients with a BMI of 30–35 kg/m^2^ and complications, endoscopic sleeve gastroplasty (ESG) as one of the strategies to achieve the desired body weight in these patients.

In summary, multimodal prehabilitation remains a promising strategy for optimizing the treatment of obese patients prior to ventral hernia surgery. While traditional strategies have primarily focused on weight loss, contemporary optimization now includes advanced methods such as GLP-1 analogues and bariatric procedures. However, the lack of standardized protocols and high study dropout rates remain significant barriers to establishing formal guidelines. Currently, high-quality randomized controlled trials (RCTs) have failed to demonstrate long-term effects in this population; clinical decisions should remain individualized, tailored to each patient’s specific risk factors, comorbidities, and functional status.

## 6. Components of Prehabilitation in Ventral Hernia

### 6.1. Smoking and the Risk of Obesity

#### 6.1.1. The Effect of Smoking on Metabolism and Inflammation

Tobacco smoking and obesity are among the major public health challenges of the 21st century. Although smoking was once believed to reduce body weight due to nicotine’s metabolic and appetite-suppressing effects, current evidence shows a more complex relationship involving not only total body mass but also fat distribution and the risk of visceral obesity.

A large Taiwanese study [100] involving 27,908 men demonstrated that smokers, especially heavy smokers, had higher BMI and waist circumference, indicating increased overall and abdominal obesity. Similar findings have been reported in European populations, including a Danish Mendelian randomisation study [11], which confirmed positive associations between smoking, BMI, WHR, and visceral fat. Numerous other studies worldwide support the link between smoking and abdominal obesity [101,102,103].

As highlighted in publication Mohapatra et al. [104], the rising popularity of e-cigarettes, particularly among young adults, introduces an additional dimension to this issue, as these devices are increasingly used both as an alternative to traditional cigarettes and as a tool for weight control. Contrary to the perceived use of e-cigarettes for weight control, a Swedish study [105] involving 3055 individuals aged 22–25 found significantly higher rates of overweight/obesity among e-cigarette users compared with non-smokers (36.7% vs. 22.3%), suggesting that electronic cigarette use may also contribute to weight gain.

These findings highlight the multifaceted metabolic effects of smoking, including altered energy expenditure, appetite regulation, and fat distribution. Increased adiposity is associated with elevated inflammatory markers, as shown in a study by Kantor et al. [106], where current smokers had significantly higher high-sensitivity C-reactive protein (hsCRP) levels than former or never smokers, reflecting chronic inflammation that may promote collagen degradation.

Research and analyses indicate that both traditional smoking and e-cigarette use significantly contribute to weight gain. Therefore, preventive strategies aimed at reducing overweight, obesity, and their associated complications should specifically target individuals who smoke.

#### 6.1.2. The Effect of Smoking on Collagen and Connective Tissue

Smoking impairs connective tissue metabolism by reducing type I and III collagen synthesis [107] and decreasing skin elasticity compared with non-smokers [108]. Although elevated hsCRP does not directly indicate collagen degradation, it reflects chronic systemic inflammation induced by cigarette smoke, which is accompanied by oxidative stress and impaired fibroblast function [109]. Oxidative stress damages the collagen-rich extracellular matrix (ECM) and promotes the activity of matrix metalloproteinases (MMPs) and proinflammatory cytokines, further accelerating collagen breakdown and inhibiting its synthesis [110].

These mechanisms collectively weaken connective tissue integrity. Thus, increased hsCRP levels in smokers and reduced collagen synthesis [107,108] likely represent parallel manifestations of inflammation-driven tissue remodelling caused by tobacco smoke. Consequently, smoking contributes to alterations in the collagen matrix and overall connective tissue structure.

Nicotine also promotes visceral fat accumulation, which is associated with insulin resistance, type 2 diabetes, dyslipidaemia, and increased cardiovascular risk [11,100]. Weight gain is frequently observed after smoking cessation, although its magnitude varies: 1.4 kg on average in the study by Krukowski et al. [111], 1.49–3.55 kg among individuals with depression [112], and 3.5 kg in postmenopausal women [113]. Weight gain has also been reported in individuals switching from traditional cigarettes to e-cigarettes [114].

Current evidence suggests that cigarette smoking and e-cigarette use may contribute to visceral fat accumulation through metabolic and inflammatory pathways. Visceral obesity increases intra-abdominal pressure and is associated with reduced connective tissue quality, potentially elevating the risk of ventral hernias. However, direct evidence linking smoking-related visceral obesity to primary ventral hernia development remains limited, indicating the need for further research.

All these metabolic and inflammatory processes negatively affect the structure of the connective tissue, which may result in defects of the fascia and abdominal wall.

#### 6.1.3. Smoking and the Risk of Ventral Hernia

Smoking is recognised as an important factor influencing the development of various hernias, postoperative recovery, and surgical outcomes. In prehabilitation for ventral hernia in patients with obesity, smoking cessation aims to enhance treatment effectiveness [2] and reduce recurrence risk [115], which is further increased by excess body weight. Cessation of smoking for 4–8 weeks prior to surgery reduces postoperative morbidity rates and limits the risk of complications in the wound healing process [99]. For epigastric and umbilical hernia repair, it is recommended to stop smoking for at least 4 to 6 weeks before surgery [116].

Nicotine promotes chronic inflammation by stimulating pro-inflammatory cytokine production, weakening connective tissue integrity and potentially increasing hernia risk. Elevated cytokine levels in smokers are well documented: Aldaham et al. [117] reported higher IL-6 concentrations in male smokers, while the Mach cohort study [118] showed significantly increased TNF-α and IL-6 levels in adipose tissue of smokers compared with never and former smokers. These findings have been confirmed in other studies [119], particularly in obese individuals and those with comorbidities [120].

Such cytokine-driven inflammation may alter adipose tissue metabolism, promoting visceral obesity and contributing to abdominal hernia formation. Visceral fat accumulation increases intra-abdominal pressure and compromises integrity of the abdominal wall structures, further predisposing individuals to hernias.

It should be emphasised that the coexistence of smoking and abdominal obesity constitutes a significant risk factor for the development of ventral hernia. While visceral adiposity leads to a chronic increase in intra-abdominal pressure, smoking-induced inflammation, reflected, among others, by elevated hsCRP levels, promotes collagen degradation. This synergistic effect compromises fascial integrity, thereby accelerating hernia formation and increasing the risk of recurrence. Therefore, in patients at risk of ventral hernia who smoke, targeted preventive strategies should be implemented.

#### 6.1.4. The Impact of Smoking on Postoperative Complications and the Importance of Quitting Smoking

Smoking is a well-established risk factor for postoperative complications and poorer surgical outcomes. Large-scale evidence confirms its negative impact: in a cohort of 169,458 patients [121], preoperative smoking was associated with higher 30-day mortality, increased wound and respiratory complications, and more cardiovascular events after abdominal hernia surgery compared with non-smokers and former smokers. Similar findings were reported by Petro et al. [122], who observed higher rates of postoperative wound infections in smokers (12.0% vs. 7.4%), mainly due to increased cellulitis (2.4% vs. 1.2%) and wound exudate (5.5% vs. 1.2%), although overall SSI rates did not differ significantly. Kudsi et al. [123] likewise found no significant difference in SSI incidence between smokers and non-smokers undergoing robotic abdominal hernia surgery.

The combined effects of smoking and obesity further increase postoperative risk. In the study by Park et al. [124], both factors independently predicted SSI after open abdominal hernia repair. The lowest SSI rate occurred in non-smokers with BMI < 24.2 kg/m^2^ (1.9%), while the highest was observed in smokers with BMI > 42.3 kg/m^2^ (12%). These findings demonstrate that rising BMI amplifies SSI risk, particularly in active smokers, underscoring the importance of smoking cessation as part of ventral hernia prehabilitation.

Smoking-related complications, such as wound infections, serous exudates, and delayed healing, prolong hospitalisation, increase healthcare costs, and reduce patient quality of life. Because smoking is a modifiable risk factor, cessation at least four weeks before surgery is recommended. Nicotine replacement therapy (NRT) is considered an effective and acceptable method to support cessation and reduce nicotine-related perioperative risks [115,125].

Despite extensive evidence linking smoking to postoperative complications, data on its role in the development of primary abdominal hernias remain limited. Future studies on obese patients with ventral hernia should evaluate postoperative outcomes and thoroughly elucidate the role of smoking in the pathogenesis of primary hernia.

### 6.2. Alcohol and the Risk of Obesity

Alcohol is rapidly metabolised and not stored in the body, which disrupts lipid oxidation and promotes abdominal fat accumulation [126]. This mechanism contributes to central obesity and related comorbidities in individuals who consume alcohol excessively. Evidence from several studies supports this association: Kazibwe et al. [127] reported that heavy drinkers had higher levels of epicardial, hepatic, visceral, and intramuscular fat compared with abstainers, while subcutaneous fat was lower. In adults with type 1 diabetes, higher alcohol intake was similarly associated with increased visceral fat mass (r^2^ = 0.23, β = 0.083, *p* = 0.04) [128]. The study by Torres et al. [126] found that excessive beer consumption increased the likelihood of elevated WC and WHR, with PR values of 1.03 (95% CI 1.00–1.07) in men and 1.10 (95% CI 1.04–1.15) in women.

Other studies [127,129,130] consistently show that alcohol consumption is a significant risk factor for visceral fat accumulation across different populations and age groups. As visceral obesity increases intra-abdominal pressure and weakens abdominal wall structures, excessive alcohol intake may indirectly contribute to a higher risk of abdominal hernias. These findings suggest that reducing alcohol consumption may support weight-management strategies and potentially lower ventral hernia risk.

#### 6.2.1. Alcohol and the Risk of Ventral Hernia

A meta-analysis on risk factors for ventral hernia development [131] identified alcohol consumption as a major contributor, although drinking frequency per week did not modify this risk. Alcohol use has also been linked to poorer postoperative outcomes. Howard et al. [132] reported that alcohol consumption was associated with longer hospital stays and higher treatment costs after incisional hernia repair. Similarly, Egholm et al. [133] showed that abstinence for 4–8 weeks before elective surgery reduced postoperative complications, although the authors noted that available studies were small and should be interpreted with caution

This means that abstinence from alcohol should be emphasized before surgical procedures in order to effectively reduce complications.

#### 6.2.2. The Effect of Alcohol on the Structure of Connective Tissue and Postoperative Complications

Direct evidence linking alcohol to primary ventral hernia formation is limited, but several mechanisms may explain this association. Alcohol impairs cutaneous wound healing [134] through weakened immune responses, increased susceptibility to infection, elevated pro-inflammatory cytokines, and tissue hypoxia. It also disrupts collagen synthesis and alters matrix metalloproteinase activity, leading to abnormal connective tissue remodelling [135]. These processes weaken fascial structures, hinder postoperative healing, and may increase the risk of both primary hernia formation and recurrence.

Given these mechanisms, alcohol consumption clearly exerts detrimental effects on tissue integrity and healing, making alcohol reduction an important component of prehabilitation in obese patients preparing for ventral hernia surgery.

### 6.3. The Role of Psychological Support in Prehabilitation Before Abdominal Hernia Surgery

Ventral hernia (VH) poses a significant psychological burden for the patient, which makes quality of life (QoL) one of the most important indicators in preoperative preparations. A range of personal, psychological, social, and environmental factors influence patients’ daily experiences and well-being. Mental health, in particular, plays a pivotal role, as it determines both the disease trajectory and final clinical outcomes [136]. Evidence supporting the effectiveness of early intervention is derived from other medical fields; for example, studies in oncological patients have shown that preoperative psychological rehabilitation significantly improves immune function [137].

Among the psychological factors contributing to poorer outcomes and reduced postoperative QoL, preoperative anxiety, depression, low self-esteem, and a perceived lack of bodily control are most frequently cited [80]. The scale of the issue is illustrated by a cohort study of elderly patients undergoing major surgery, which identified a high prevalence of comorbidities: anxiety (*n* = 11,836; 36.4%), depression (*n* = 11,258; 34.6%), and psychosis (*n* = 1924; 5.9%) [14]. Importantly, patients who underwent a preoperative mental health assessment were less likely to experience postoperative complications.

In the specific population of patients with abdominal hernia, Smith et al. [138] highlighted a particular intensity of feelings such as shame, shock, guilt, negative body image, and difficulties in adaptation. Consequently, psychological intervention has become an indispensable component of prehabilitation programmes. Psychological support, including preoperative telephone consultations, was a key component of the prehabilitation programme studied by Boukili et al. [139]. This intervention effectively improved patients’ mental well-being by reducing anxiety and depression while enhancing various quality-of-life indicators. The efficacy of a multimodal approach was further confirmed by Villa et al. [13]; in a group of 1126 patients prior to VHR, the application of cognitive-behavioural therapy, relaxation techniques, mindfulness-based interventions, hypnosis, self-help coping strategies, and narrative medicine significantly alleviated both anxiety and pain.

Understanding a patient’s emotional needs is therefore crucial in the surgical preparation process [138]. In this context, the psychologist is an essential member of the interdisciplinary team delivering prehabilitation [80]. Professional support allows for the modification of surgical stress, which directly impacts wound healing, immune system efficiency, pain perception, and the patient’s overall mood during recovery [13].

We therefore recommend that psychological intervention be included as a component of prehabilitation for patients with ventral hernia and obesity, as it may modulate the physiological response to surgical stress. Preoperative optimisation of mental health, particularly addressing anxiety, depression, and body image disturbances, has been shown to correlate with improved immune function, reduced pain perception, and a lower incidence of postoperative complications. Accordingly, psychological support should be incorporated into standard prehabilitation protocols for ventral hernia repair to ensure better clinical outcomes and long-term patient satisfaction.

### 6.4. The Impact of Physical Activity on Obesity and Visceral Fat

Obesity, particularly the accumulation of visceral fat, is often associated with reduced physical activity and impaired functional capacity. Regular physical activity plays a central role in weight management by reducing visceral adipose tissue (VAT) and improving metabolic markers, including blood glucose, glycated haemoglobin, and lipid profiles [140,141,142,143,144]. These benefits are supported by reductions in pro-inflammatory cytokines, including IL-6, as demonstrated in structured exercise interventions [145].

#### 6.4.1. The Impact of Different Forms of Physical Activity on Visceral Fat Reduction and Obesity

Randomised clinical trials and meta-analyses indicate that regular aerobic exercise (≥150 min/week) in overweight or obese individuals significantly reduces waist circumference, total fat mass, and visceral adipose tissue (VAT) [142,146]. Combined aerobic and resistance training provides additional benefits in improving body composition and further reducing total fat mass.

Meta-analyses comparing exercise modalities show that aerobic exercise remains most effective for weight loss, while combined training is particularly beneficial for reducing body mass index, waist circumference, and pro-inflammatory markers such as IL-6 and TNF-α [147]. While endurance training reduces CRP, IL-6, and TNF-α, resistance training has been shown to increase anti-inflammatory IL-10; notably, combined exercise amplifies the reduction in TNF-α [148]. These shifts in inflammatory profiles are generally accompanied by reductions in fat mass, highlighting the synergistic effects of multimodal exercise [149,150].

Overall, the evidence suggests that regular physical activity, particularly aerobic and combined modalities, effectively reduces visceral fat and modulates inflammatory responses. This is of particular relevance for patients preparing for abdominal hernia surgery, where VAT reduction and inflammation control may significantly improve surgical outcomes.

#### 6.4.2. Physical Activity as a Component of Prehabilitation in Ventral Hernia Repair

Physical activity in patients prior to ventral hernia reconstruction can significantly improve their quality of life and physical function. This was demonstrated in a study by Berkvens et al. [151], in which the intervention comprised a three-month supervised exercise programme, including cardiovascular, strength, and respiratory muscle training, overseen by a physiotherapist. Following the intervention, nine men and two women reported improvements in both physical function and health-related quality of life (HRQoL). Consequently, all patients successfully underwent reconstruction with fascial closure.

In the context of obese patients, prehabilitation is considered an essential component of therapy, although research findings in this area are mixed. Prehabilitation, incorporating physical activity and weight management interventions, has been proposed as a means to improve treatment outcomes in obese patients with ventral hernia. In a study by Bernardi et al. [97], patients with a BMI of 30–40 kg/m^2^ were assigned to a prehabilitation group, which included diet and exercise, with surgery contingent on achieving ≥7% weight loss or no weight gain over six months. Although prehabilitation programmes improved short-term health indicators, after two years there was no significant difference in the proportion of patients free from recurrence or complications between the intervention and standard care groups.

In the study by Renshaw et al. [12], the relationship between physical activity levels and the risk of postoperative complications was assessed in 2994 patients undergoing ventral hernia repair (VHR). Based on self-reported activity levels, four groups were identified: inactive (no exercise), occasional (once a month), moderate (once a week), and vigorous (more than once a week). The analysis demonstrated that increased frequency of physical activity was significantly associated with a reduced risk of postoperative complications (OR: 0.70; *p* = 0.008 for occasional activity; OR: 0.62; *p* = 0.006 for moderate activity; OR: 0.67; *p* = 0.04 for vigorous activity). Similar associations were observed regarding hospital readmissions, with physical activity significantly reducing the risk of readmission (OR: 0.04 for occasional activity; OR: 0.40 for moderate activity; OR: 0.03 for vigorous activity; *p* = 0.01). It is important to note that physical activity level was not associated with length of hospital stay, changes in quality of life, or postoperative pain intensity.

These findings suggest that regular preoperative physical activity in patients undergoing VHR may reduce the risk of complications and rehospitalisation. However, it does not appear to significantly influence the length of hospital stay or patient-reported quality of life.

#### 6.4.3. Recommendations and Current Guidelines for Physical Activity in Ventral Hernia Repair

Currently, there are no clear EHS recommendations regarding physical activity before and after VHR. During the 41st Congress of the European Hernia Society (EHS), a survey of surgeons [152] assessed recommendations for postoperative activity. More than 50% of respondents considered a two-week restriction of strenuous physical activity sufficient following laparoscopic procedures. After open procedures (laparotomy) or ventral hernia repair with mesh (including the IPOM technique), most experts recommended four weeks of activity restriction. In complex hernia repair techniques, such as component separation, 37% of experts considered four weeks to be the minimum recommended rest period.

Danish recommendations suggest an immediate return to daily activities, while sports or weightlifting should be avoided for 2–4 weeks after surgery [153]. Reviews and guidelines [98] support prehabilitation as a strategy for risk factor modification; however, they emphasise that current evidence on the role of physical activity remains inconsistent, limiting the development of formal recommendations. There are also no precise guidelines regarding the type and intensity of exercise before and after abdominal wall repair [154].

Available data suggest that physical activity as a component of prehabilitation may improve short-term outcomes after VHR; however, evidence regarding long-term effects remains limited. Further well-designed studies are required to determine the optimal type, intensity, and duration of exercise in this patient population.

In conclusion, physical activity appears to be a beneficial component of multimodal prehabilitation in patients undergoing ventral hernia repair. Nevertheless, the heterogeneity of available studies and the lack of high-quality long-term data highlight the need for large, randomised controlled trials to establish evidence-based exercise protocols.

### 6.5. Weight Loss Before Abdominal Hernia Surgery

Effective preoperative preparation for abdominal hernia repair is based on weight optimization, primarily through structured lifestyle changes, as recommended by the latest EHS consensus [62].

Joint recommendations from the EHS and American Hernia Society (AHS) recommend reducing BMI to <35 kg/m^2^ before surgery in obese patients [116]. According to the 2025 EHS guidelines, weight loss should be achieved through dietary interventions, with consideration of bariatric surgery and/or GLP-1 analogues depending on the severity of overweight or obesity [62]. The EHS systematic review within the “Prehabilitation” project [93] demonstrated that smoking cessation and weight loss may reduce postoperative complications.

In a study by Holland et al. [155], the mean BMI decreased from 38.2 to 34.0 kg/m^2^ before surgery. At long-term follow-up, the average weight loss was approximately 11 kg, and 70% of patients maintained at least half of the weight lost. In contrast, a review by Marcolin et al. [156], including five studies (*n* = 465), found no significant differences between patients with and without prehabilitation in terms of hernia recurrence (OR 0.66), seroma, haematoma, wound infection, or overall complications.

The Weight Management Navigator (WMN) programme described by Maskal et al. [157] represents a potential tool to support preoperative preparation. However, its effectiveness was limited, with only 9 of 191 eligible individuals completing the programme. Mean weight loss was 5.97 kg in the intervention group compared with 1.8 kg in the control group (*p* = 0.01). These findings suggest potential benefit, although further multicentre studies are required to assess effectiveness and cost-effectiveness.

Given the complexity of this patient population, an interdisciplinary approach involving surgeons, dietitians, physiotherapists, and psychologists is recommended to optimize weight loss and improve surgical outcomes. Such a multidisciplinary approach enables a more comprehensive understanding of the complexity of obesity, particularly in patients with a history of bariatric surgery.

#### GLP-1 Receptor Agonists as a Novel Strategy in Prehabilitation of Ventral Hernia

A novel weight loss strategy for patients with hernias involves pharmacological support, including GLP-1 receptor agonists. Current EHS treatment algorithms propose the use of GLP-1 analogues as part of the preoperative preparation process for obese patients prior to VHR. Specifically, these algorithms suggest GLP-1 therapy for individuals with a BMI of 25–30 kg/m^2^ with comorbidities, those with a BMI of 30–35 kg/m^2^, and all patients with a BMI > 35 kg/m^2^ [62]. This class of medications appears to accelerate preoperative weight loss in obese patients without increasing the risk of postoperative complications.

These observations are supported by a retrospective study [158] involving 70 patients with various hernia types (abdominal, incisional, umbilical, parastomal, lateral, and inguinal). Following treatment with GLP-1 agonists, the mean preoperative BMI reduction was 5.3 ± 3.5 kg/m^2^. In a subgroup followed for 6 months post-surgery (*n* = 7), the weight loss was maintained (mean BMI at surgery: 29.0 ± 2.8 kg/m^2^ vs. BMI at 6 months: 28.5 ± 3.4 kg/m^2^; *p* = 0.46). Furthermore, the 30-day postoperative complication rate was 9.1%, with a reoperation rate of 6.1% and a hernia recurrence rate of 3.0% (*n* = 1).

In another retrospective review [159], patients achieving preoperative weight loss via GLP-1 agonists combined with lifestyle modifications were compared to a control group using lifestyle modifications alone. The mean percentage weight loss (14.9 ± 7.5 kg vs. 12.4 ± 6.9 kg; *p* = 0.39) and mean BMI reduction (6.0 ± 3.8 kg/m^2^ vs. 4.9 ± 2.3 kg/m^2^; *p* = 0.43) were comparable between the groups. Notably, there was no significant difference in 30-day morbidity (8.3% vs. 27.3%; *p* = 0.13). The efficacy of GLP-1 in this context was further corroborated by Remulla et al. [160], who enrolled 258 patients with a BMI of 40–55 kg/m^2^ in a 6-month prehabilitation programme. This randomised trial suggested a significant potential benefit of pharmacological weight loss prior to elective surgery.

Furthermore, Romain et al. [161] demonstrated the positive impact of GLP-1 agonists on clinical outcomes in patients undergoing incisional hernia repair (IHR). The mean percentage weight loss ranged from 7.4% to 11.3% in the GLP-1 group. Although not statistically significant, the overall rate of postoperative complications was lower in the GLP-1 cohort compared to the control group (45.8% vs. 59.6%; *p* = 0.2).

Current evidence suggests that weight loss achieved through GLP-1 agonists in hernia patients is comparable to that achieved via conventional methods (diet, lifestyle changes) and does not adversely affect surgical safety. In individuals with obesity, GLP-1 analogues may be considered a useful tool for weight loss. However, it should be noted that most available evidence comes from retrospective or pilot studies, and high-quality randomised data remain limited. Therefore, although GLP-1 agonists represent a valuable component of multimodal pharmacological prehabilitation in obese patients undergoing hernia surgery, further research is required to optimize clinical protocol.

### 6.6. The Influence of Diet on the Development of Hernia and Obesity

#### 6.6.1. The Association Between a Pro-Inflammatory Dietary Pattern and the Development of Obesity

An increasing number of studies indicate that a diet with a high pro-inflammatory potential, measured by the DII^®^ or its energy-adjusted version (E-DII), promotes the development of visceral obesity as well as metabolic and cardiovascular disorders. Higher DII^®^ values correlate with an increased risk of overweight, visceral obesity, metabolic syndrome, hypertension, hyperglycaemia, and hypertriglyceridaemia in adolescents and adults with normal BMI.

A study in Tehran (*n* = 535) reported a positive correlation between higher DII^®^ and the risk of overweight and obesity in adolescent boys [162]. In the prospective NHANES III cohort (*n* = 3521, BMI 18.5–24.9 kg/m^2^), higher DII^®^ was associated with an increased risk of cardiovascular mortality in individuals with visceral obesity, which intensified with higher WHR, without affecting cancer-related mortality [163]. In the Multiethnic Cohort study (*n* > 215,000), higher E-DII was associated with greater total body fat, visceral adipose tissue (VAT), and liver fat, and strongly negatively correlated with HEI-2010 (r = −0.78) [164].

An analysis of 2359 cardiology patients showed that the highest DII tertile was associated with larger waist circumference (*p* = 0.013), higher systolic (*p* = 0.044) and diastolic (*p* = 0.014) blood pressure, an adverse lipid profile, and more cardiovascular events [165]. In northern China (*n* = 1936, >55 years), a pro-inflammatory diet increased the risk of hyperglycaemia (*p* = 0.03), hypertension (*p* = 0.03), hypertriglyceridaemia (*p* = 0.02), and abdominal obesity (*p* = 0.01) [166]. Analysis of over 3900 individuals (~60 years) showed a positive correlation of visceral obesity with palmitic acid levels and stearoyl-CoA and Δ6 desaturase activity, and a negative correlation with linoleic acid, α-linolenic acid, DHA, and Δ5 desaturase activity [167].

The cited studies and their findings indicate that a pro-inflammatory diet significantly increases the risk of visceral obesity, metabolic syndrome, and cardiovascular complications, even in individuals with a normal BMI. Conversely, a diet rich in vegetables, fruits, fibre, flavonoids, zinc, magnesium, selenium, and polyunsaturated fatty acids exerts a protective effect, emphasising the pivotal role of diet in preventing metabolic and cardiovascular diseases.

#### 6.6.2. The Role of Dietary Factors, Processed Foods, and Simple Sugars in the Pathogenesis of Obesity

Both central obesity and abdominal or inguinal hernias may be associated with diet quality and lifestyle. A prospective case–control study (*n* = 203) found that higher consumption of red meat (*p* < 0.001) and bread (*p* = 0.011), as well as low fibre intake (*p* = 0.004), were significantly linked to an increased risk of inguinal hernia. Smoking, alcohol consumption, and severe constipation were also identified as significant risk factors [168]. These findings suggest that dietary modification, including reducing meat intake and increasing vegetable consumption, may help prevent hernias, particularly in individuals with central obesity.

The six-year Seniors-ENRICA-1 study demonstrated that high intake of ultra-processed foods (UPF) significantly increased the risk of central obesity in older adults; individuals with the highest UPF consumption had over a 60% higher risk compared to those with the lowest intake, independent of fibre intake and adherence to the Mediterranean diet [169]. Similar associations were observed in a Brazilian cohort of dialysis patients (*n* > 1000), where higher UPF consumption, male sex, lower educational attainment, and insufficient physical activity were linked to increased central obesity prevalence (>77%) [170]. Biological mechanisms include the high energy density of UPF, low nutritional value (low in fibre, vitamins, and antioxidants), and high content of saturated fats, trans fatty acids, and refined sugars, promoting chronic inflammation, metabolic disturbances, and weakened muscle structures.

In the CARDIA study (participants aged 18–30, 30-year follow-up), higher added sugar intake was significantly associated with weight gain (mean + 2.3 kg, *p* = 0.01), increased waist circumference (+2.2 cm, *p* = 0.005), and higher risk of general (HR 1.28) and abdominal obesity (HR 1.27) [171]. These data indicate that added sugars in food products may substantially contribute to the pathogenesis of central obesity.

Research shows that a diet high in processed foods and simple sugars significantly contributes to obesity, which is a risk factor for abdominal hernias. Limiting these food groups is therefore recommended to minimize the risk.

#### 6.6.3. Diet and Ventral Hernia Risk

The impact of different types of food products on the development of ventral hernia is not yet fully known. One of the few studies by Yang et al. [131] used the Mendelian randomisation (MR) method to examine the relationship between various dietary factors and the risk of different types of hernias, including ventral hernia. The results of this review showed that the frequency of alcohol consumption increased the risk of ventral hernia (OR ≈ 1.309; *p* ≈ 0.0477). The consumption of cooked vegetables was also associated with an increased risk of ventral hernia (OR ≈ 4.475; *p* ≈ 0.0380). A lower risk of ventral hernia was observed in the case of cheese and dried fruit consumption (respectively OR ≈ 0.434; *p* ≈ 0.000536 and OR ≈ 0.322; *p* ≈ 0.00716). However, the authors emphasise the limitations of the analysis, including the small sample size and the specificity of the population. Another, the only available study from Turkey [168], examined the relationship between dietary factors, hernias, and constipation. The results showed that a high intake of red meat and chicken, high bread consumption, as well as low fibre intake and low-energy diets were associated with the occurrence of inguinal hernia. Despite a growing number of studies, the relationship between specific dietary components and the occurrence of hernias remains unclear.

Research suggests that diet, primarily through the consumption of foods such as cheese and cooked vegetables, contributes to the development of abdominal hernias. However, this association requires further randomized clinical trials. It should also be noted that future studies should examine the dietary habits of people diagnosed with abdominal hernias, with the aim of potentially improving their eating habits and identifying foods that may contribute to the development of this condition.

Table 2 Summarises the key studies analysing the impact of diet on the development of central obesity and ventral hernias.

### 6.7. The Importance of Dietary Intervention in Obesity with Associated Ventral Hernia

In obese patients preparing for ventral hernia surgery, nutritional intervention should focus on controlling total energy intake while ensuring an adequate protein supply to preserve lean muscle mass and prevent catabolism. Maintaining muscle mass is particularly important in the perioperative period, as it supports physical function, wound healing, and postoperative recovery. Dietary strategies should therefore combine caloric restriction with sufficient protein intake and be individually tailored to the patient’s functional capacity, comorbidities, and physical limitations [82].

In overweight and obese patients, preoperative weight loss is recommended to reduce the risk of perioperative and postoperative complications [172,173]. Evidence suggests that the most favourable outcomes are achieved when the preoperative preparation period lasts 3–12 months, with an optimal duration of 6–12 months. No single dietary approach is universally suitable for obese patients, particularly when obesity coexists with ventral hernia and other chronic conditions. Nutritional care should be individualised, prioritising both weight reduction and adequate intake of essential nutrients, vitamins, and minerals. Effective dietary intervention should focus on high-quality foods and the promotion of sustainable, healthy eating habits [174].

A properly planned dietary intervention not only reduces the risk of complications but can also improve metabolic outcomes and shorten hospitalization time.

#### 6.7.1. Protein Before Ventral Hernia Surgery

Adequate protein intake is fundamental to muscle mass preservation, effective wound repair, and robust immune function. Patients (especially before major abdominal surgery) are recommended to consume up to 1.5 g/kg of ideal body weight (IBW) [175] taking into account lean body mass [173]. The increased protein requirement results from the need to protect against malnutrition, including protein malnutrition, to which patients are particularly susceptible after surgical procedures [176,177,178] and in ventral and hiatal hernias [64,179]. This is significant because the presence of preoperative protein malnutrition has been shown to significantly increase the incidence of complications and worsen the postoperative condition [180]. Given that abdominal wall integrity and muscle strength are key determinants of surgical outcomes in ventral hernia repair, adequate perioperative protein intake may be of particular importance in this patient population.

Casein and whey are the main groups of proteins found in milk and contain all common amino acids, including essential ones [181]. The digestion of these two fractions is described as “fast” and “slow.” The casein fraction coagulates in the acidic environment of the stomach, which delays its emptying and induces a slow postprandial rise in plasma amino acids. Whey, on the other hand, induces a rapid, high, and transient increase in plasma amino acids. For this reason, whey can be considered to have more satiating properties [182].

Several studies have examined the effects of whey protein supplementation on body weight, insulin metabolism, and lipid profile. In a 12-week study, Pal et al. [181] compared whey supplementation with casein and a control group in overweight and obese individuals. Whey significantly decreased total cholesterol and LDL cholesterol compared with the casein group (*p* = 0.026 and 0.045) and control group (*p* < 0.001 and 0.003), and fasting insulin levels were also significantly reduced (*p* = 0.049 and 0.034). Regarding glucose metabolism, Allerton et al. [183] showed that consumption of 20 g of whey 15 min before breakfast reduced the postprandial glucose peak by 17%, and during the meal by 9%. Other studies reported similar reductions in postprandial glucose [184,185]. These results indicate that whey protein acts as an effective metabolic modulator, potentially improving preoperative glycemic control and lipid metabolism in obese patients before surgery.

Whey protein supplementation has also been shown to be associated with improved lean body mass recovery following surgery in obese individuals [186]. These results suggest that this particular protein fraction may also be a critical factor in preserving functional muscle mass postoperatively.

Clinical data specifically addressing protein intake in ventral hernia repair (VHR) remain scarce. In a study by Crocetti et al. [187], the effect of a high-protein diet on abdominal muscle remodelling in 45 patients undergoing elective abdominal wall reconstruction (AWR) was assessed. Patients were divided into two groups: Group A received a standard diet (men: 2300 kcal, 103 g protein; women: 1800 kcal, 80 g protein) from one month before to three months after surgery, while Group B received the same diet plus 34 g of purified protein daily. No significant difference in muscle thickness was observed at three months (10% vs. 19%, not significant), but at six months, Group B showed significantly greater rectus abdominis thickening (13% vs. 32%, *p* < 0.05), indicating a positive effect of protein supplementation.

In a similar study of patients with colon cancer, Gillis et al. [188] evaluated the effect of an 8-week perioperative intervention with whey protein. Preoperatively, the whey protein group demonstrated significant improvement in functional walking capacity (+20.8 m) compared with the placebo group (+1.2 m), although this trend did not reach statistical significance (*p* = 0.27). Postoperatively, recovery rates at four weeks were similar in both cohorts (*p* = 0.81). As the cited study showed, an effective improvement in functional walking capacity was achieved before surgery with whey protein supplementation. However, the result turned out to be not statistically significant, so further research is recommended. Studies conducted mainly on cancer patients indicate that whey protein supplementation contributes to improved muscle strength and reduces postoperative complications [189,190].

Although available studies suggest that whey protein supplementation in the perioperative period can improve glycaemic control, body composition, and muscle regeneration, the current state of knowledge is mainly based on small clinical trials, often involving patients with various disease entities (obesity, cancer, abdominal wall reconstruction). The results are heterogeneous, and some analyses did not achieve statistical significance, despite observed trends beneficial for whey supplementation.

In the context of ventral hernia surgery, the evidence is particularly limited and includes small groups of patients and a short observation period, which does not allow for a clear formulation of clinical recommendations. There is also a lack of large, statistically powerful randomised studies that would assess the effect of whey intake on postoperative complications, hospital stay, or long-term treatment outcomes in patients with ventral hernias. Therefore, currently, whey protein supplementation can be treated as a promising strategy but one that requires further verification in well-designed studies.

#### 6.7.2. Protein-Enriched Mediterranean Diet (PEMD)

For many years, the Mediterranean diet (MD) has been recognised as one of the healthiest dietary models in the world. Its effectiveness has been confirmed in reducing cellular insulin sensitivity, oxidative stress, and inflammation. This translates into a reduced risk of neurodegenerative [191,192,193], cardiovascular diseases, and diabetes, which has been confirmed by numerous prospective and randomised studies [194,195,196,197]. For this reason, the MD has become a model for many international dietary recommendations.

Low-calorie Mediterranean diets with a balanced macronutrient composition are recommended by current guidelines for weight loss [198,199]. One variant of the Mediterranean diet that has been used for weight reduction in patients in recent years is the protein-enriched Mediterranean diet (PEMD). A modified protein-enriched Mediterranean diet was used by participants in one study [200] that included participants with diabetes and pre-diabetes. Subjects followed a Mediterranean diet supplemented with high-quality whey protein and engaged in physical activity for 16 weeks. In the pre-diabetes and type 2 diabetes groups, fasting plasma glucose decreased by 12.0 mg/dL and 19.6 mg/dL, respectively, reaching normal and pre-diabetic levels. In the type 2 diabetes group, HbA1c improved from 6.8% to 6.0%, reaching pre-diabetic levels. Overweight participants with normal metabolism experienced reductions in body weight and improved body composition. These findings suggest that a protein-rich Mediterranean diet can improve glycaemic control and support metabolic health in obese patients

Gastaldo et al. [201] evaluated an 8-week protein-enriched Mediterranean diet (PEMD) in 37 obese patients (BMI ≥ 40 kg/m^2^) undergoing LSG. The diet (30% protein, 25% fat, 45% carbohydrates) significantly reduced body weight (−16.7%, *p* = 0.01), visceral fat (−27.4%), liver volume (−29.1% and −25.2%), and fat mass (−14.1%), without affecting fat-free mass (*p* = 0.0960), while also improving liver enzymes and lipid profiles. Similar benefits were reported by Schiavo et al. [202].

A comparison of a high-protein diet and a Mediterranean diet in obese patients showed that both improve fasting insulin resistance, with the high-protein diet having a stronger effect on the glycaemic profile [203]. Similarly, a diet described as low-carbohydrate/high-protein is an effective short-term way to lose weight in obese patients, as shown in a study comparing this diet with MD [198].

Because insulin resistance and metabolic disorders have an inflammatory basis and often coexist with obesity and VH (ventral hernia), the use of PEMD with a reduced energy intake may be a promising nutritional strategy in preparing obese individuals for bariatric surgery or VH surgery. However, it should be emphasised that the effectiveness of this diet, especially in the context of VH, requires randomised clinical trials to assess its real impact on obese patients in the preoperative period.

#### 6.7.3. Low-Energy and Very Low-Energy Diets (LED and VLED) for Preoperative Weight Loss in Surgery

Low-Energy Diet (LED) and Very Low-Energy Diet (VLED) are meal replacements designed for significant and rapid weight loss. These diets provide 0.8–1.5 g of protein per kg of ideal body weight (IBW) Additionally, they are characterised by a restricted content of carbohydrates and fat (≤80 g carbohydrates/day and 15 g fat/day). The energy value of these diets is usually in the range of 800–1200 kcal/day, with most protocols being based on 800 kcal. These diets are most often prescribed for a period of 4 to 12 weeks [204].

##### The Effectiveness of LED/VLED in Obese Patients

Both the LED and VLEDs are most commonly used in patients prior to bariatric surgery. Most available clinical trials demonstrating the effectiveness of the LED and VLED in weight loss come from bariatric surgery studies and specifically focus on the VLED [205,206,207]. A systematic review of 1868 patients [208], who used both LED (800–1200 kcal/d) and VLED (450-<800 kcal/day), showed that both of these diets lead to short-term weight reduction. In one of the RCT reviews [209], an analysis was performed on 8 studies with a total of 862 patients who followed LED/VLED protocols. About half of the patients achieved a weight reduction of ≥5%. In other studies and meta-analyses [210,211,212,213], the effectiveness of LED and VLED in weight reduction in obese patients before surgical procedures was also demonstrated, as well as a reduction in liver volume by 5 to 20% [214].

In addition to preoperative weight loss, dietary interventions such as the VLED in the period immediately preceding surgery have other positive effects, including reducing liver volume, reducing visceral fat, and reducing inflammation. These effects can lead to an increased surgical space, which improves visibility and facilitates the procedure [210].

This effect of VLED is currently documented in a very small number of studies. A study by Griffin et al. [9] compared a control group (without dietary intervention or physical activity implementation) with a group that used a VLCD diet and physical activity for 2–12 weeks before surgery. The study group consisted of obese patients (BMI ≥ 30 kg/m^2^) awaiting procedures, including ventral hernia repair. As a result, the group on the VLCD diet showed a greater reduction in body weight (−5.5 kg for VLCD compared to −0.9 kg for the control group, *p* < 0.05), a reduction in waist circumference (−6.6 cm for VLCD compared to 0.6 kg for the control group, *p* < 0.05), and a lower number of complications after 30 days (*n* = 4/21) than the control group (*p* > 0.05). The same author [215] in a similar study also showed that a VLCD diet used before surgery in obese individuals reduced the risk of surgical site infection, rehospitalisation, and cardiac incidents (*p* < 0.05).

The use of low-energy diets (LED and VLED) in the preoperative period is an effective strategy for optimizing the management of obese patients. Their role extends beyond mere weight loss. Their clinical significance primarily lies in reducing liver volume and visceral fat, which directly translates into improved technical performance during surgery. Scientific evidence, including studies conducted directly in the population of patients with ventral hernias (VHR), confirms that a brief, supervised VLED intervention significantly reduces the risk of surgical site infections (SSIs) and cardiovascular events. Therefore, these protocols can be considered an integral element of multidisciplinary rehabilitation for patients with a high BMI.

##### Practical Recommendations for Preoperative Care and the Use of Low-Energy Diets

The optimisation of nutrition before surgery, also in the VLCD diet scheme, should primarily be based on an appropriate ratio of proteins, fats, carbohydrates, and micronutrients, which play a key role in preparing the patient for surgery. The recommended intake of complete protein in obese individuals should be in the range of 1.2–1.5 g/kg of ideal body weight (IBW) per day, and its source should be easily digestible whey protein. Liquid protein supplements can be products that support LED and VLED. Carbohydrate intake should be at a level of 40–50% of the total energy requirement, with a particular focus on complex carbohydrates such as whole-grain products, legumes, and vegetables. Fats should constitute 20–30% of the total energy intake. In the daily diet of individuals before surgical procedures, special attention should be paid to those from fish oil, olive oil, avocado, nuts, and seeds, as they provide essential unsaturated fatty acids, which are responsible for, among other things, reducing inflammation. Consuming an adequate amount of essential micronutrients, such as iron, calcium, vitamin D, and B vitamins, in obese individuals before surgery is crucial to prevent their deficiencies, especially in the postoperative period [175].

A systematic review of studies on the use of LED/VLED in the preoperative period, mainly in bariatric surgery, confirms their effectiveness in short-term weight reduction. Although most data comes from the population of patients preparing for bariatric surgery, metabolic mechanisms, including the reduction in visceral fat tissue, are also important in patients before ventral hernia surgery. Although the effectiveness of low-energy diets (LED) and very low-energy diets (VLED) in short-term weight reduction is widely documented, the scientific evidence regarding the validity of using such diets remains limited and ambiguous. This highlights the need for more randomized controlled trials and the development of a standardized weight loss protocol for surgical procedures, particularly for hernias.

#### 6.7.4. Modifications to VLCD in Prehabilitation for Obese Patients

##### VLCD with Protein Supplementation

The effectiveness of a VLCD diet supplemented with additional protein was assessed in the study by Jo et al. [216]. In this study, obese patients participated in a 12-week weight loss program using a patented very low calorie diet (Optifast^®^), with an energy value of 1120 kcal/day.

Participants were assigned to either a Standard Treatment Control (CON, *n* = 5) or Resistance Training (RT, *n* = 6) group for 14 weeks. Both groups experienced significant reductions in total body mass (CON: 19.4 ± 2.3 kg; RT: 15.8 ± 1.5 kg) and fat mass (CON: 14.7 ± 1.8 kg; RT: 15.1 ± 2.1 kg), with no differences between groups. Resistance training helped preserve fat-free mass while not impeding overall body or fat mass loss. These interventions also led to improvements in resting metabolism and muscle function in men and women with morbid obesity undergoing a VLCD programme with protein supplementation.

Nutritional interventions using LED/VLED, especially when combined with high-quality protein, effectively reduce body weight and body fat, improve metabolic parameters, and support the preservation of muscle mass in obese patients undergoing surgery. Therefore, LED/VLED enriched with protein and appropriately tailored physical activity represent a promising strategy for the rehabilitation of obese patients undergoing surgery. However, it is recommended that future randomized trials be designed to standardize protocols and confirm long-term clinical benefits.

##### VLCKD—Characteristics and Efficacy

Another modification of the VLCD is the VLCKD (Very Low Carbohydrate Ketogenic Diet). Unlike the classic VLCD, VLCKD is characterised by an extremely low carbohydrate intake and a dominance of fats. Due to its restrictive nature, once the target weight is reached, it is recommended to adopt a balanced lifestyle for long-term maintenance of weight loss effects. The VLCKD diet may be effective not only in weight reduction but also in improving lipid indicators [217], glycaemic control [217,218], and even in achieving remission of type 2 diabetes (T2DM) [219,220,221]. In a study by Albanese et al. [222], 72 patients followed a VLCKD diet and 106 followed a VLCD diet. Baseline BMI was 46.3 ± 6.3 kg/m^2^ in the VLCKD group and 43.1 ± 6.9 kg/m^2^ in the VLCD group; immediately preoperatively, BMI was 43.9 ± 5.9 kg/m^2^ and 41.9 ± 6.8 kg/m^2^, respectively. Postoperative haemoglobin correlated with diet-induced BMI reduction (141.2 ± 75.8 vs. 190.7 ± 183.6 mL, *p* = 0.032; 13.1 ± 1.2 vs. 12.7 ± 1.5 g/L, *p* = 0.04). Fewer patients in the VLCKD group required a hospital stay >3 days (2.8% vs. 10.4%, *p* = 0.048), indicating superior postoperative outcomes compared with VLCD. This experience shows that VLCKD demonstrated better results than VLCD in terms of postoperative outcomes, affecting post-surgery haemoglobin levels and length of hospital stay. In a study by Balestra et al. [223], participants were placed on a VLCKD diet. Its effect on weight reduction and liver markers was studied over 8 weeks of its use. After this time, a decrease in the body weight of the study participants and liver parameters was observed. Many other studies also indicate the effectiveness of this diet in obese individuals in reducing body weight, visceral fat, and liver parameters, as well as its volume [214,224,225].

Studies have shown that the VLCKD (very low-carbohydrate ketogenic diet) effectively reduces body weight and adipose tissue, including visceral fat, improves metabolic parameters such as lipid profile and glycemic control, and may also shorten hospital stays and have a beneficial effect on postoperative hemoglobin levels.

Although the VLCKD diet is a promising strategy for preoperative nutritional intervention in obese patients, due to the restrictive nature of the diet, further research and long-term monitoring are necessary to maintain its effects and assess its safety.

##### Results of the Effectiveness of VLCD and VLCKD Diets and Practical Recommendations

The use of VLCKD and VLCD diets, conducted under the supervision of a clinical dietitian, seems to be a promising form of prehabilitation for obese patients planning ventral hernia surgery. It is worth noting that similar dietary strategies are successfully used in bariatric surgery, where they contribute to, among other things, a reduction in liver volume and improved patient health safety. Although these interventions are effective in short-term weight reduction, their long-term effectiveness depends on maintaining lifestyle changes after the procedure. However, the improper use of low-energy diets by patients can lead to a deficiency of minerals and vitamins [226], which, especially in the perioperative period, can result in adverse health complications. Therefore, further research is needed to determine the optimal duration and composition of such diets, as well as their impact on outcomes in hernia surgery. Due to the complexity of the problem, an interdisciplinary approach involving surgeons, clinical dietitians, and other specialists seems crucial for improving the safety and effectiveness of interventions in patients with obesity and a hernia.

Although the described VLCD and VLCKD diets are effective for short-term weight loss, evidence of their effectiveness in patients with abdominal hernia remains limited. In summary, optimising nutritional status, with particular emphasis on protein intake and appropriate dietary strategies, can improve the safety and outcomes of surgical treatment. However, scientific evidence specifically relevant to patients with ventral hernia is limited, and current recommendations are based primarily on extrapolation from bariatric and oncological surgery. Therefore, further well-designed randomised controlled trials are necessary to develop consistent and effective nutritional protocols for this patient population.

### 6.8. Omega-3 Fatty Acids and Vitamin D in VHR Prehabilitation in Obese Patients

#### 6.8.1. Omega-3 Fatty Acids in Prehabilitation in Obese Patients

Eicosapentaenoic acid (EPA) and docosahexaenoic acid (DHA), found in fish, fish oils, nuts, and seeds, are essential dietary components with well-documented anti-inflammatory properties. They modulate cytokine expression, support muscle mass maintenance, and act as precursors of specialised pro-resolving mediators (SPMs), such as resolvins, protectins, and maresins, which promote the resolution of inflammation and tissue repair [227].

In obese individuals, omega-3 fatty acid supplementation has been shown to reduce triglyceride levels, lower systolic and diastolic blood pressure, and improve insulin sensitivity, thereby potentially reducing cardiovascular risk [228,229,230]. Grytten et al. [231] studied adults aged 30–70 years with abdominal obesity who received 3–4 g/day of EPA/DHA (fish oil) or 15–20 g/day of linoleic acid (safflower oil), finding that omega-3 supplementation significantly reduced inflammatory markers. Similarly, Ruiz-Tovar et al. [232] reported that omega-3 use in bariatric patients led to greater preoperative weight loss, reduced postoperative pain, and lower C-reactive protein levels. These studies highlight the anti-inflammatory and perioperative benefits of omega-3 supplementation in obese patients.

Omega-3 fatty acids may represent an important component of prehabilitation in obese patients due to their anti-inflammatory effects. Supplementation has been associated with improvements in inflammatory markers, metabolic parameters, and selected postoperative outcomes. Therefore, they may be particularly beneficial in patients with obesity.

#### 6.8.2. The Effectiveness of Omega-3 Fatty Acids in Perioperative Management

In a study by Sorensen et al. [233], patients undergoing colorectal cancer surgery received EPA and DHA supplementation for seven days both before and after their procedure. The results showed no significant differences in postoperative complications (*p* = 0.544), the risk of disease recurrence at three years (RR 1.66, 95% CI 0.65–4.26), or five-year survival rates (69.2% vs. 81.7%, *p* = 0.193). Evidence from other trials, however, suggests potential benefits of omega-3 fatty acids. In cardiac surgery, fish oil supplementation was associated with a reduced risk of major perioperative and postoperative cardiovascular events [234]. Meta-analyses also indicate that omega-3 fatty acids may enhance immune function and lower the risk of postoperative complications [235].

Nevertheless, findings regarding perioperative omega-3 supplementation remains inconsistent. Some studies report no significant impact on postoperative C-reactive protein (CRP) levels, while the effect on cytokines, including tumour necrosis factor-alpha, interleukin (IL)-6, and IL-10 appears equivocal [236]. In contrast, when administered as part of parenteral nutrition, omega-3 fatty acids have been shown to reduce infection rates, shorten intensive care unit and hospital stays, and lower inflammatory markers [237].

As research demonstrates, current evidence suggests that the impact of omega-3 fatty acids on surgical outcomes is heterogeneous. This further highlights the need for well-designed, randomised controlled trials to clarify their clinical role and help establish standardised supplementation dosages.

#### 6.8.3. Omega-3 Fatty Acids in Ventral Hernia Repair

Data regarding the role of omega-3 fatty acids in ventral hernia repair (VHR) remain scarce. However, interesting insights have emerged from the use of omega-3-coated meshes. In a study by Yücel et al. [238], researchers observed reduced infection rates and fewer adhesions, which potentially contribute to improved wound healing and lower recurrence rates. Conversely, Kong et al. [239] noted that such coatings might actually induce unexpected inflammatory reactions, leading the authors to advise caution.

Despite these conflicting reports, the established anti-inflammatory and metabolic benefits of omega-3 fatty acids make them a promising component of perioperative nutritional strategies, particularly given their positive impact on lipid profiles. Nevertheless, transitioning from experimental mesh coatings to standardised oral supplementation in VHR patients will require more focused studies to confirm both the safety and clinical efficacy in this specific population.

### 6.9. Vitamin D in VHR Prehabilitation and Perioperative Care

Vitamin D supplementation before ventral hernia repair can provide significant benefits both before and after surgery. Its role in regulating the immune system, modulating inflammation, and musculoskeletal function significantly supports the maintenance of adequate vitamin D levels. This can accelerate wound healing, reduce postoperative complications, and aid recovery, particularly in high-risk groups [240,241,242].

A study conducted by AlMulhim et al. [243] among obese women undergoing laparoscopic ventral hernia repair found that supplementing with 5000 IU of cholecalciferol and 1000 mg of calcium daily led to weight loss, which was associated with improved surgical outcomes and a lower risk of complications. Furthermore, studies conducted in both animal models and humans suggest that vitamin D may contribute to reducing the severity of postoperative peritoneal adhesions, potentially reducing complications and improving outcomes [244,245]. Vitamin D also plays a key role in postoperative wound healing, influencing tissue regeneration and modulating inflammatory responses [246,247]. Supplementation at doses of 1000 and 3000 IU/day, in particular, may improve wound healing and reduce scar thickness. In this regard, one study found that a daily dose of 3000 IU demonstrated better results than 1000 IU [242]. Vitamin D deficiency may also hinder wound healing [248,249] and increase the risk of complications, including infections [250]. Importantly, the literature indicates that low vitamin D levels predispose patients to a higher risk of developing surgical site infections (SSI) [251].

This has been particularly observed in obese patients undergoing bariatric surgery, for whom vitamin D status assessment and appropriate supplementation are recommended due to the high prevalence of vitamin D deficiency in this population both before and after surgery [252,253,254].

Although existing studies suggest a beneficial effect of vitamin D supplementation on treatment outcomes, wound healing, and the risk of complications after ventral hernia repair, the number of studies in this area remains limited. Furthermore, well-designed studies with adequate population sizes are needed to definitively determine the optimal doses and the effect of supplementation on postoperative outcomes in patients with ventral hernia and obesity. Nevertheless, monitoring vitamin D levels and ensuring adequate supplementation should be an integral part of patient preparation for ventral hernia surgery, particularly in populations at risk of deficiency.

Omega-3 fatty acids and vitamin D play an important role in modulating inflammation, supporting immune function, and promoting wound healing in patients undergoing surgery. Available research suggests their potential benefits, primarily in abdominal surgery, especially in obese individuals. Studies specifically addressing ventral hernia repair are still lacking. Current findings suggest the need for further, well-designed randomised controlled trials to determine optimal supplementation strategies and confirm clinical efficacy. Nevertheless, targeted nutritional optimisation, including assessment and monitoring of omega-3 and vitamin D levels, should be considered an integral part of patient preparation for ventral hernia repair.

## 7. Prehabilitation for Patients with Ventral Hernias: Practical Recommendation

Due to the incomplete recommendations regarding preoperative management in patients with obesity and abdominal hernia, the following recommendations have been proposed (Table 3).

## 8. The DEPP Concept: A Clinical Prehabilitation Algorithm

**Figure 3:**
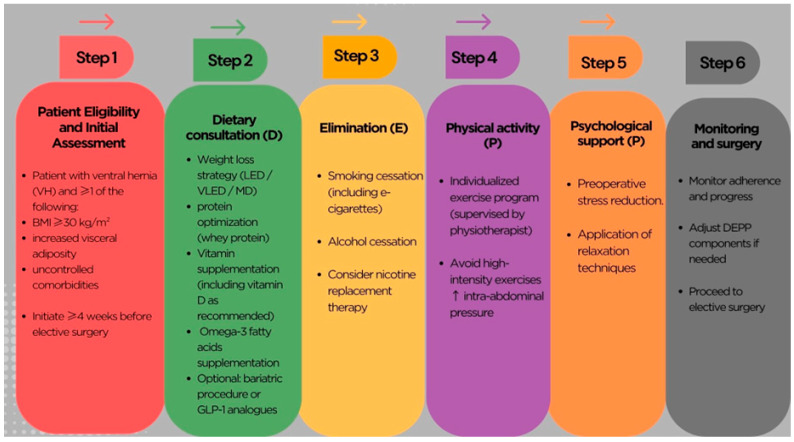
Clinical algorithm for prehabilitation of patients with obesity and ventral hernia.

The ESPEN recommendations [84] may provide a basis for developing new guidelines for prehabilitation in patients with VHR preparing for surgery, with particular emphasis on the role of nutrition and supplementation, substance abuse cessation, and physical activity. Based on a review of the literature and available research, we propose the DEPP prehabilitation algorithm (dietary consultation, elimination, physical activity, and psychological support) for patients undergoing ventral hernia surgery and with obesity (Figure 3). The algorithm was developed to systematize the clinical approach and determine the steps to be taken in the treatment of patients with obesity and abdominal hernia. The DEPP algorithm was developed based on articles demonstrating the positive effects of individual components of prehabilitation in patients with obesity and abdominal hernia, the latest recommendations [2,9,14,30,62,96,98,153,175,201,204] and studies showing that patients with abdominal hernia experience deterioration of their mental health. The DEPP algorithm was also partially developed based on our clinical experience and observations, primarily involving dietary consultations and physical activity. The DEPP algorithm consists of the following prehabilitation stages:

**STEP 1: Patient Eligibility and Initial Assessment**—Upon diagnosis of an abdominal hernia in patients with overweight and obesity (especially BMI > 30 kg/m^2^) or increased visceral adiposity, patients are considered eligible for prehabilitation. Stabilization of comorbidities, such as diabetes or hypertension, is recommended before starting the program. Prehabilitation should commence at least four weeks prior to elective abdominal hernia repair.
**STEP 2: Dietary Consultation (D)**—The consultation should be conducted by a clinical dietitian and include weight reduction, selection of an appropriate dietary intervention, and supplementation with vitamin D, omega-3 fatty acids, and protein, preferably whey protein. At this stage, consideration of a bariatric procedure or pharmacological GLP-1 receptor agonists may also be appropriate if clinically indicated. Bariatric procedures, such as endoscopic sleeve gastroplasty (ESG), may be considered in selected patients with ventral hernia if technically feasible and after individual risk assessment, in accordance with EHS recommendations [62]. Considering preoperative weight loss highlights its importance in preoperative optimization. Patients should be encouraged to achieve clinically meaningful weight loss, as even modest weight reduction may improve surgical outcomes.
**STEP 3: Elimination (E)**—This stage involves smoking cessation, including discontinuation of electronic cigarette use, and cessation of alcohol consumption. Intervention should be implemented after a dietary consultation with a dietitian. Alternatively, nicotine replacement therapy may be considered.
**STEP 4: Physical Activity (P)**—Recommended under the supervision of a physiotherapist, with exercises appropriately tailored to the patient’s physical capabilities. The intervention should be implemented after a dietary consultation and elimination of smoking and alcohol.
**STEP 5: Psychological Support (P)**—This stage involves the individualised selection of appropriate psychological interventions by a qualified clinical psychologist, contributing to the reduction in preoperative stress in the patient. Psychological consultation should be considered before a planned procedure.
**STEP 6: Monitoring and surgery:** This stage involves regular assessment of the patient’s progress. If necessary, the components of the DEPP algorithm should be adjusted throughout the prehabilitation process. Once the predefined goals are achieved, the patient may proceed to elective surgery.

It is recommended that the above prehabilitation elements be used simultaneously, but they can also be implemented gradually, depending on the patient’s individual needs and abilities. In our experience, this approach increases patient engagement before surgery.

The DEPP model can also serve as a comprehensive and practical tool for preoperative preparation of obese patients, not only those undergoing VHR but also other procedures, including bariatric surgery.

## 9. Limitations

This review has several limitations that should be considered when interpreting the results. Firstly, the available literature regarding prehabilitation in patients with obesity and ventral hernia is characterised by significant heterogeneity. Included studies vary in design, population characteristics, sample sizes, and the type and duration of interventions, which limits direct comparability and the ability to draw definitive conclusions. Secondly, there is a scarcity of high-quality evidence, particularly randomised controlled trials (RCTs). Much of the available data stems from observational studies or indirect evidence, which may introduce bias and weaken the overall strength of the conclusions. Furthermore, many studies focus on individual components of prehabilitation rather than a comprehensive, multimodal approach. The narrative nature of this review is an additional limitation, as it does not employ a formal systematic methodology or quantitative synthesis. While every effort was made to include relevant and current literature, the selection process may be subject to inherent bias.

Moreover, the proposed DEPP algorithm is based on a synthesis of available evidence, clinical reasoning, and our own observations, rather than validated prospective trials. Therefore, its clinical utility and efficacy require further validation through RCTs.

Finally, prehabilitation in the context of abdominal hernia surgery for patients with obesity is a nascent and evolving field. There is a clear need for well-designed RCTs that comprehensively evaluate all elements of prehabilitation, including dietary intervention, lifestyle modification, physical activity, and psychological support.

## 10. Conclusions

Prehabilitation, including a multimodal approach, should be considered an essential element of patient preparation, especially for obese patients undergoing ventral hernia surgery. It may support preoperative optimisation and contribute to improved postoperative outcomes, particularly in patients with higher BMI and comorbidities. Various models are currently being developed and implemented in international centres. However, discrepancies between them reflect the limited availability of high-quality scientific evidence and the lack of standardised protocols. Accordingly, the recommendations and algorithm presented in this study are based in part on the authors’ clinical experience and indirect evidence, and should not be interpreted as a validated clinical protocol, but rather as a preliminary clinical framework for the management of this specific patient population.

Although the current results are promising, further confirmation of the effectiveness and safety of this approach requires well-designed, high-quality randomised controlled trials. Future research should focus on long-term outcomes and comprehensive evaluation of all components of prehabilitation.

## Figures and Tables

**Table 1:** Summary of landmark studies included in the review.

Step of DEEP	Intervention Category	Key Study & Year	Study Type	Sample (*n*)	Key Results & Clinical Outcome	LoE *
**D—Diet**	VLCD & Nutrition (inc. Protein/Vit D)	Griffin et al. (2024) [9]	RCT	51	Preoperative VLCD reduces body weight and waist circumference	II
		Abdehgah et al. (2020) [10]	Prospective	300	Preoperative Vit D deficiency (<20 ng/mL) is a strong predictor of SSI.	III
**E—Elimination**	Smoking Cessation	Carrasquilla et al. (2024) [11]	Mendelian Rand.	>400,000	Causal link: Smoking impairs collagen synthesis and increases abdominal obesity.	I
**E—Exercise**	Physical Activity	Renshaw et al. (2021) [12]	Observational	2994	Preoperative exercise reduces length of stay (LOS) and wound complications.	III
**P—Psychological**	Mental Support	Villa et al. (2020) [13]	Systematic Review	9 studies	Psychological prep significantly reduces pre-op anxiety and post-op pain.	I
		Khalil et al. (2025) [14]	Cohort	32,543	Clinical psychology consultation improves patient’s readiness for major surgery.	III

* LoE: I—Meta-analysis/Systematic Review; II—RCT; III—Cohort/Prospective Study/Observational; *n*, sample size; RCT, Randomized Controlled Trial; SSI, Surgical Site Infection; VLCD, Very Low-Calorie Diet; Vit D, Vitamin D.

**Table 2:** Summary of studies analysing the impact of diet on the development of central obesity and ventral hernia.

Reference (Year)	Dietary Components	Study Group	Impact on the Body
Vahid et al. (2020) [162]	DII > 0.02	adolescent boys aged 12 to 16 years	↑ body weight, BMI, body fat mass, ↓ muscle mass
Choi et al. (2023) [163]	DII	adults aged 20 to 90 years with normal BMI	↑ risk of death from cardiovascular disease in people with abdominal obesity
Lozano et al. (2022) [164]	DII	adults aged 60 to 77 years	↑ total body fat, VAT, and liver fat content
da Silva et al. (2021) [165]	DII	patients with established cardiovascular events	↑ waist circumference, SBP and DBP, total cholesterol, and number of cardiovascular events
Li et al. (2022) [166]	DII	elderly over 55 years	↑ risk of MetS, hyperglycaemia, TAG concentration, hypertension, and abdominal obesity
Alsharari et al. (2017) [167]	↑ palmitic acid, ↑ SCD; ↑ linoleic acid, α-linolenic acid, DHA, ↑ Δ5-desaturase activity	adults, mean age ~60 years	SFA profile ↑ abdominal obesity; PUFA (LA, ALA, DHA) ↓ central obesity
Idiz et al. (2020) [168]	↑ intake of red meat, bread, and ↓ intake of fibre	18–80-year-old patients	↑ risk of inguinal hernia
Sandoval-Insausti (2020) [169]	↑ intake of UPF	participants aged 60 and over	↑ risk of abdominal obesity
Endy et al. (2024) [171]	↑ added sugar consumption	black and white women and men, aged 18–30 years	↑ weight gain, waist circumference, risk of developing general obesity and abdominal obesity
Yang et al. (2024) [131]	consumption of alcohol or cooked vegetables	-	↑ risk of ventral hernia
	consumption of cheese or dried fruit	-	↓ risk of ventral hernia

DII, Dietary Inflammatory Index; BMI, Body Mass Index; VAT, visceral adipose tissue; SBP, systolic blood pressure; DBP, diastolic blood pressure; MetS, metabolic syndrome; TAG, triglyceride; UPF, ultra-processed foods; ↑, increase; ↓, decrease.

**Table 3:** Authors’ proposed practical recommendations for prehabilitation for patients with VH and obesity.

Recommendation Category	Detailed Guidance for the Prehabilitation of Patients with Obesity and Ventral Hernia
Patient Selection	Individuals with BMI ≥ 30 kg/m^2^, with particular emphasis on those with high visceral adiposity.
Duration	Typically 4–6 weeks, adjustable based on weight loss rate and metabolic stabilisation.
Composition of the multidisciplinary team	Surgeon and others, if required, clinical dietitian, psychologist, physiotherapist
Core Components	Health optimisation (metabolic control), nutritional intervention, tailored physical activity, psychological support.
Weight Loss Strategies	Body weight reduction to the maximum extent achievable. Consideration of GLP-1 receptor agonists or bariatric surgery, based on individual clinical indications.

## Data Availability

No new data were created or analyzed in this study.
